# Irregular Baseline Brain Activity in Coronary Artery Disease Patients with Cognitive Impairment: A Resting-state Functional Magnetic Resonance Imaging Study

**DOI:** 10.2174/1567202619666220516124552

**Published:** 2022-11-25

**Authors:** Jingchen Zhang, Jueyue Yan, Jianhua Niu, Zhipeng Xu, Xing Fang, Jingyu You, Tong Li

**Affiliations:** 1 Department of Critical Care Medicine, The First Affiliated Hospital, Zhejiang University School of Medicine, Zhejiang, China

**Keywords:** Coronary artery disease, functional magnetci resonance imaging,cognition, low-frequency fluctuations, mini-mental state examination, montreal cognitive assessment

## Abstract

**Objective:**

Cognitive impairment has been suggested to be associated with coronary artery disease [CAD]; however, the underlying mechanism is not fully understood. Our current study aimed to explore the brain activity in CAD patients compared to healthy controls [HCs].

**Methods:**

Twenty-two CAD patients and 23 HCs were enrolled in our study. A low-frequency oscillation at the voxel level in all participants based on the amplitude of low-frequency fluctuations [ALFF] was measured using resting-state functional magnetic resonance imaging. All participants underwent neuropsychological examinations [Mini-Mental State Examination, MMSE and Montreal Cognitive Assessment, MoCA] and visual acuity examination.

**Results:**

CAD patients showed significantly lower ALFF values [*P* < 0.05] in the right precuneus gyrus [Precuneus_R], left supramarginal gyrus [Supramarginal_L], left angular gyrus [Angular_L], and left middle cingulum gyrus [Cingulum_Mid_L] than healthy controls. Lower MoCA scores in CAD patients significantly correlated with lower Supramarginal_L [*P* = 0.001] and Cingulate_Mid_L [*P* = 0.004] ALFF values. Reduced visual acuity significantly correlated with lower Precuneus_R [*P* = 0.019] and Cingulate_Mid_L [*P* = 0.011] ALFF values in CAD patients.

**Conclusion:**

These findings may provide further insight into the underlying neuropathophysiology of CAD with cognitive impairment.

## 1 INTRODUCTION

An increase in the elderly population has resulted in a higher incidence of cardiovascular disease, and due to treatment options, patients live longer [1]. Coronary artery disease [CAD], the commonest cardiovascular disease, occurs in 43% of males and 41% of females over 81 years. CAD shares mutual risk factors with cerebrovascular disease, such as age, gender, and history of cardiovascular disease [2].

CAD has been reported to cause embolic stroke and chronic cerebral hypoperfusion, which may lead to cognitive impairment [3-5]. Reports have shown that CAD is associated with an increased risk for cognitive impairment and dementia [4, 6]. It has been suggested that CAD causes cerebral small vessel disease, which leads to degeneration of cerebral structures and may affect cognitive function [7]. Although recent reports have demonstrated a link between CAD and impaired brain function, very little is known about the cerebral functional activity which occurs during CAD.

Resting-state functional magnetic resonance imaging [rs-MRI] is an important imaging modality that allows researchers and clinicians to explore the neuropathophysiology of a disease. Rs-MRI is relatively easy to perform since it simply requires patients to remain still with their eyes closed. Therefore, the technique has a wide range of potential applications in clinical studies [8, 9]. Quantitative measurement of low-frequency oscillation [LFO] amplitude offers another potentially useful tool for the detection of synchronization of LFO between spatially distinct brain regions [10]. The amplitude of low-frequency fluctuation [ALFF], which measures the total power of a given time course within a specific frequency range such as 0.01 – 0.08 Hz, has been used to examine local spontaneous patterns in the resting state [10].

ALFF analysis has been used to explore the functional modulations and showed the pathophysiological characteristics in the resting state of patients with cognitive impairment [11]. Given the neurological and cognitive deficits shown in previous CAD reports [7], investigation of the activity changes in the brain may provide a clue for the cerebral changes that occur in CAD. Our current report aimed to analyze the characteristics of ALFF from rs-MRI data and reveal changes in brain activity in CAD patients.

## 2 METHODS

This observational cross-sectional study was done at the First Affiliated Hospital of Zhejiang University School of Medicine. The inclusion criteria for CAD patients were as follows: 1). age between 35 and 80 years; 2). diagnosed with CAD; 3). could cooperate during magnetic resonance imaging.

The control group involved individuals who attended our hospital for annual health check-ups and had no history of neurologic or cardiovascular diseases.

All participants were evaluated for cardiovascular risk factors, medical history, and medication use and had a comprehensive cardiovascular physical examination by a cardiologist. The study was approved by the Ethics Committee of First Affiliated Hospital of Zhejiang University School of Medicine. Participants recruited provided written informed consent before enrolling in the study.

### 2.1 Neuropsychological Examinations

All participants underwent a Montreal Cognitive Assessment, MoCA, and Mini-Mental State Examination, MMSE, which are examinations to screen for cognitive decline. These examinations have a total score of 30, and a score lower than 26 indicates worse cognition in MoCA, while a score lower than 24 indicates worse cognition in MMSE.

Visual acuity examination for both eyes was done for all participants. Visual acuity for both eyes was later converted to the minimum angle of resolution [LogMAR] for data analysis.

## 2.2 MAGNETIC RESONANCE IMAGING PROTOCOL

Whole-brain MRI data were acquired at the Center for Brain Imaging Science and Technology, First Affiliated Hospital of Zhejiang University School, on a Siemens MAGNETOM Prisma 3T scanner [Siemens, Erlangen, Germany]. All participants were placed in the machine with foam padding around the head to reduce motion; they were asked to keep still with their eyes closed during imaging.

An echo-planar imaging sequence was used to acquire the functional images with the following parameters: 60 axial slices, thickness/gap = 2.0/0mm, in-plane resolution = 64 x 64, repetition time [TR] = 2000ms, echo time [TE] = 34 ms, flip angle = 62° and field of view [FOV] = 220 x 220 mm^2^. Anatomical T1-weighted whole brain magnetization-prepared rapid gradient echo images were obtained using the following parameters: 160 sagittal slices, slice thickness/gap = 1.2/0 mm, in-plane resolution = 512 x 512, TR = 5000 ms, TE = 2.9 ms, inversion time [TI] = 700 ms, flip angle = 4° and FOV = 256 x 256 mm^2^.

### 2.3 Processing of MRI Data

SPM8 [http://www.fil.ion.ucl.ac.uk/spm] was used to implement pre-processing of all fMRI data while data processing was done with Data Processing Assistant for Resting-State fMRI [http://www.restfrmi.net]. The initial 10 volumes of the functional images were discarded to remove initial transient effects and to allow the participant to adjust to the scanner noise before pre-processing. The rest of the fMRI images were acquired with slice timing for the acquisition delay between slices and correction of head motion. All participants who were under imaging had less than 1.5 mm maximum displacement in x, y, or z and 1.5° angular motion during imaging. Spatial normalization and resampling to 3 mm voxels were used to acquire realigned images, while a Gaussian filter [6 mm FWHM] was used to spatially smoothen the images. Smoothened images were filtered using a typical temporal bandpass [0.01 – 0.08 Hz] to reduce low-frequency drift, and physiological high-frequency respiratory and cardiac noise. Linear trends were removed within each time series. Lastly, spurious variances from several sources were removed by linear regression, including six head motion parameters, along with average signals from cerebrospinal fluid and white matter.

### 2.4 Calculation of ALFF

REST software [http://www.restfmri.net] was used to calculate the ALFF. The preprocessed time series were first converted to a frequency domain with a fast Fourier transform, and the power spectrum was obtained. The square root of the power spectrum was calculated for each frequency of the power spectrum, and the averaged square root was obtained across 0.01 – 0.08 Hz at each voxel [10]. The average square root was taken as the ALFF at the given voxel and standardized by dividing the whole brain voxel average ALFF, which measures the absolute strength or intensity of spontaneous LFO.

### 2.5 Statistical Analysis

Differences in ALFF between the two groups were assessed with a second-level random-effect two-sample t-test on the individualized normalized ALFF maps in a voxel-by-voxel manner. With the small sample size enrolled, we performed nonparametric statistical tests between the ALFF maps of the two groups using the Statistical NonParametric Mapping toolbox [http://warwick.ac.uk/snpm] to confirm the results between the two groups. AlphaSim, a Monte Carlo cluster-wise simulation program implemented in AFNI [http://afni.nimh.nih.gov], was used to correct for multiple comparisons [12] and to protect against false positives. The statistical threshold was set at *P* < 0.01 with a cluster size > 40 voxels, which corresponded to *P* < 0.05. All coordinates are reported in Montreal Neurological Institute coordinates as used by SPM. To investigate the correlation between ALFF and clinical implications, multivariate linear regression was used while adjusting for age, gender, hypertension, and educational level. *P* < 0.05 was considered statistically significant.

## 3 RESULTS

Twenty-two CAD patients [mean age = 59.91 ± 6.82 years, 22.73% females] and 23 healthy controls [mean age = 60.64 ± 6.79 years, 30.43% females] were included in our data analysis. Out of the 22 CAD patients, 15 [68.18%] had hypertension, 2 [9.09%] had diabetes and 4 [18.18%] had dyslipidemia. There was no significant difference in the years of education between CAD and healthy controls [*P* > 0.05, Table **1**]. Importantly, CAD patients showed significantly lower [*P* < 0.001, Table **1**] MMSE and MoCA scores than healthy controls.

### 3.1 Differences in ALFF Values Between CAD and HC

**Fig. (1):**
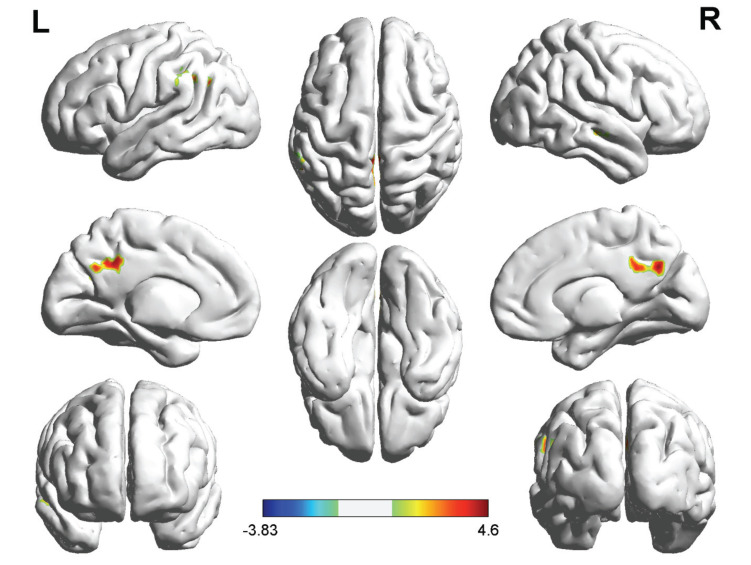
Brain regions showing ALFF differences in CAD patients and healthy controls. CAD patients showed significantly lower ALFF values in the right precuneus gyrus [Precuneus_**R**], left supramarginal gyrus [Supramarginal_**L**], left angular gyrus [Angular_L], and left middle cingulum gyrus [Cingulum_Mid_L] compared to healthy controls.

CAD patients showed significantly lower ALFF values *P* < 0.005, (Table **2**, Fig. **1**) in the right precuneus gyrus [Precuneus_R], left supramarginal gyrus [Supramarginal_L], left angular gyrus [Angular_L], and left middle cingulum gyrus [Cingulum_Mid_L] than healthy controls.

### 3.2 Correlation Between ALFF Values and Clinical Implications in CAD Patients

Lower MoCA scores in CAD patients significantly correlated with lower Supramarginal_L [*P* = 0.001, Table **3**] and Cingulate_Mid_L [*P* = 0.004, Table **3**] ALFF values. Reduced visual acuity significantly correlated with lower Precuneus_R [*P* = 0.019, Table **3**] and Cingulate_Mid_L [*P* = 0.011, Table **3**] ALFF values in CAD patients.

## 4 DISCUSSION

To the best of our knowledge, this is the first study to investigate the LFO amplitude changes in CAD patients compared to healthy controls. Compared to healthy controls, CAD patients showed significantly lower ALFF values in the right precuneus gyrus [Precuneus_R], left supramarginal gyrus [Supramarginal_L], left angular gyrus [Angular_L], and left middle cingulum gyrus [Cingulate_Mid_L]. Importantly, we showed that the lower ALFF values in some regions of the brain significantly correlated with their clinical insinuations.

Cerebral functional impairment [decreased ALFF values] predominantly occurred in the frontal, temporal and parietal lobes. Previous cerebral microstructural reports showed reduced volume in some gyri of the parietal, frontal and temporal lobe in CAD patients compared to healthy controls [13, 14]. Besides, previous neuroimaging reports also showed CAD patients to have thinner gray matter and white matter volume compared to healthy controls [15, 16]. The cortical areas linked to CAD in our present study are involved in autonomic functions [left middle cingulate gyrus] [17] and cognitive domains, such as recollection of memory [right precuneus gyrus][18], language skills [left angular gyrus] [19], and phonological processing [left supramarginal gyrus] [20]. The parietal lobe [precuneus gyrus, left supramarginal gyrus, and left angular gyrus] and frontal lobe [left middle cingulate gyrus] are regions that have been reported to be vulnerable to vascular diseases [21, 22]. Our study showed that CAD patients had significantly lower ALFF values in the right precuneus gyrus, left supramarginal gyrus, left angular gyrus, and left middle cingulate gyrus than healthy controls. Since CAD is strongly related to hypertension [23, 24], which has been reported to be linked with significant microstructural changes in both the frontal and parietal lobes [25], our report suggests that CAD may lead to a neural network dysfunction in the brain. Therein, these significant LFO changes in these areas provide insights into the cognitive changes that occur in CAD patients. Importantly, these cortical regions have a connection with memory function in humans [26], thus highlighting the association with cognition as previously reported [16, 27].

The association between lower MoCA scores and reduced ALFF values in the left supramarginal gyrus and left middle cingulate gyrus in CAD patients is in line with previous neuroimaging reports [7, 16, 28]. These reports suggest that atherosclerosis from CAD causes cerebrovascular pathology, such as white matter lesions, which are indicative of cognitive impairment. Importantly, these regions have major connections with the medial temporal lobes, which play a significant role in the successful retrieval of memory [29]. The positive correlation between ALFF values and cognitive impairment in CAD patients may suggest that reduced neural functional modulations in the regions may affect the cognition of CAD patients.

Existing studies [30-33] have confirmed that people with cardiovascular disease may be at a higher risk of developing certain types of eye problems, especially visual loss. Our study showed that visual impairment in CAD patients significantly correlated with lower right precuneus and left middle cingulate gyrus ALFF values. Previous neuroimaging reports [15, 18] have shown significant changes in these areas of the brain, indicating that neurodegeneration may occur in these areas of CAD patients. Although visual loss has been suggested to be associated with CAD, the association between the lower ALFF values suggests that neural functional amplitude changes in these areas have affected a CAD patient’s vision.

Our study has several limitations. As with most imaging tools, participant cooperation is necessary. Patient movement can diminish the quality of images and 3 participants were excluded from the study because of movement during MR imaging. LFO amplitude methodology is often studied in larger populations; larger study sample sizes are needed to confirm the importance of our results. The clinical importance of the MRI procedure was evaluated with visual acuity and cognitive tools; further studies are warranted to assess its value in treatment response and scoring systems for coronary angiography. Performing cognitive assessment could be challenging during the acute phase of CAD, as some patients could be prone to acute confusional states. Nonetheless, our study excluded patients with confusional states and delirium. Further studies may include such patients to provide a clearer view of the structural changes in the brain and their association with cognitive tools. Importantly, our cognitive assessment was limited to MoCA and MMSE; further studies with extensive neuropsychological evaluation may be needed.

## CONCLUSION

In conclusion, we used the ALFF approach derived from rs-MRI to assess the low-frequency oscillations at the voxel in CAD patients compared to healthy controls. Our report showed that CAD had reduced ALFF values in the right precuneus gyrus, left supramarginal gyrus, left angular gyrus, and left middle cingulum gyrus than healthy controls. We also showed that the lower ALFF values in CAD patients correlated with their lower MoCA scores and reduced visual acuity. These findings may provide further insight into the underlying neuropathophysiology of CAD patients.

## Figures and Tables

**Table 1:** Demographic and clinical information of study participants.

**Variable**	**CAD [n=22]**	**HC [n=23]**
-	-	-
Demographic information		
Gender [F/M]	5/17	7/16
Age [years], mean [SD]	59.91 ± 6.82	60.64 ± 6.79
Hypertension, No.	15	12
Diabetes mellitus, No.	2	1
Dyslipidemia, No.	4	5
Smokers, No.	11	9
Education, years	5.09 ± 1.41	5.11 ± 1.01
MMSE score	20.45 ± 4.01	26.73 ± 1.74
MoCA score	17.18 ± 3.13	28.18 ± 1.33
Visual acuity, LogMAR	0.13 ± 0.10	-0.14 ± 0.35

**Table 2:** Brain regions with significantly decreased ALFF value in CAD patients compared to healthy controls.

**Brain Regions**	**Voxels**	**BA**	**MNI coordinates**			***T* Value**
			**X**	**Y**	**Z**	
Precuneus_R	25	31	6	-60	33	<0.005
SupraMarginal_L	9	40	-54	-45	33	<0.005
Angular_L	23	39	-42	-63	33	<0.005
Cingulum_Mid_L	18	31	-6	-51	36	<0.005

**Table 3:** Association between ALFF parameters and clinical parameters.

**Variable**	**MMSE^Ϯ^**		**MoCA^Ϯ^**		**Visual Acuity**	
	**β Coefficient** **[95% CI]**	***P* Value**	**β Coefficient** **[95% CI]**	***P* Value**	**β Coefficient** **[95% CI]**	***P* Value**
Precuneus	0.009 [-0.015 - 0.033]	0.468	0.006 [-0.024 - 0.036]	0.697	-1.217 [-2.234 - -0.201]	0.019
Supramarginal	-0.018 [-0.051 – 0.041]	0.113	0.040 [-0.064 – 0.015]	0.001	0.223 [-0.880 – 1.326]	0.692
Angular	0.035 [-0.004 - 0.075]	0.081	0.008 [-0.044 - 0.060]	0.772	-0.829 [-2.746 – 1.087]	0.396
Cingulum	0.017 [-0.011 - 0.046]	0.235	0.045 [0.014 - 0.076]	0.004	-1.552 [-2.745 - 0.360]	0.011

**Note**: ^Ϯ^ adjusted for age, hypertension, years of education, and gender.

## Data Availability

The data that support the findings of this study are available within the article.

## LIST OF ABBREVIATIONS

CAD: Coronary Artery Disease
HC: Healthy Controls
ALFF: Amplitude of Low-Frequency Fluctuations
MMSE: Mini-Mental State Examination
MoCA: Montreal Cognitive Assessment
Precuneus_R: Right Precuneus Gyrus
Supramarginal_L: Left Supramarginal Gyrus
Angular_L: Left Angular Gyrus
Cingulum_Mid_L: Left Middle Cingulum Gyrus
rs-MRI: Resting-state Magnetic Resonance Imaging
LFO: Low-Frequency Oscillation
